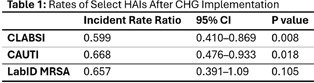# The Impact of CHG Bathing on Healthcare Associated Infections Across a Rural Hospital System

**DOI:** 10.1017/ash.2025.286

**Published:** 2025-09-24

**Authors:** Hunter Ratliff, Matthew Lokant, Janina-Marie Huss, Michael Edmond, Michael Stevens

**Affiliations:** 1 West Virginia University Health System; 2 West Virginia University; 3 WVU Medicine; 4 WVU Medicine; 5 West Virginia University

## Abstract

**Background:** We aimed to examine the impact of daily bathing with chlorhexidine gluconate (CHG) on central line associated bloodstream infections (CLABSIs), catheter associated urinary tract infections (CAUTIs), and bloodstream infections with methicillin-resistant Staphylococcus aureus (LabID MRSA) across a large, rural healthcare system. This healthcare system encompasses 8 large community hospitals, one academic hospital, and 11 hospitals with 50 or fewer beds. Starting in August 2023, all facilities were required to adopt daily CHG bathing for patients with central lines and/or in intensive care units. Some facilities also chose to adopt CHG daily bathing for patients with foley catheters. **Methods:** We analyzed the hospital-wide monthly incidence of select healthcare associated infections (HAIs) in the year before and after implementation of CHG bathing across a large, decentralized, rural healthcare system. We conducted negative binomial regressions to examine the difference in HAIs before/after implementation of CHG bathing, and we used the National Healthcare Safety Network’s (NHSN) predicted numbers of HAIs to adjust for confounding among hospitals. **Results:** After adjusting for each hospital’s predicted number of infections, we saw a 40.1% decrease in CLABSIs (p=0.008) and a 33.2% reduction in CAUTIs (p=0.018, Table 1); we also observed a 34.3% reduction in LabID MRSA, although this was not statistically significant (p=0.105). **Conclusion:** System-wide implementation of CHG daily bathing in a large, decentralized, rural healthcare system was associated with a significant reduction in CLABSIs and CAUTIs.

**Figure:**
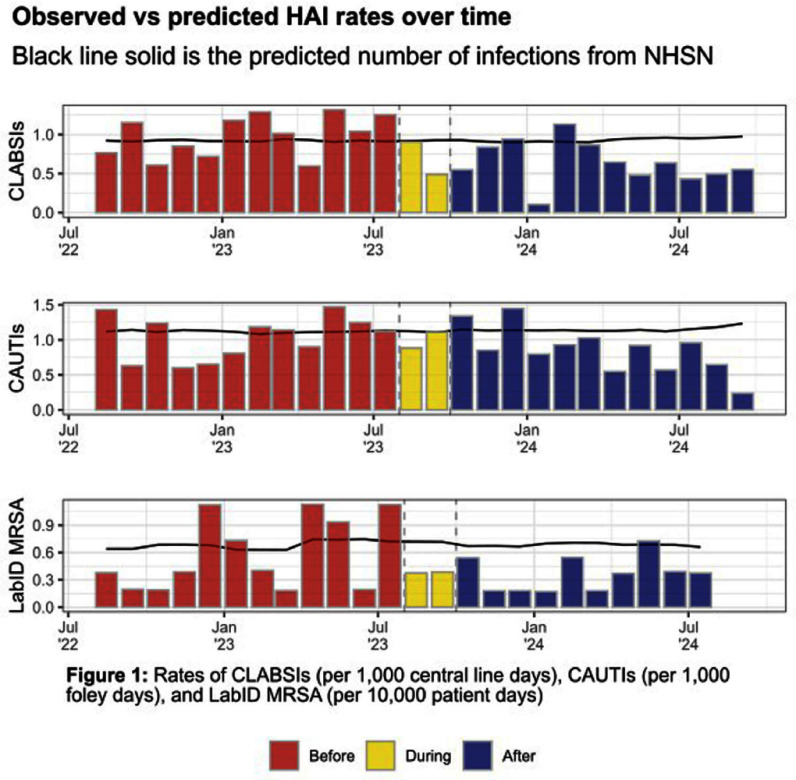

**Figure:**